# Skin lesions revealing a loco-regional and systemic metallosis

**DOI:** 10.1016/j.jdcr.2026.03.047

**Published:** 2026-04-07

**Authors:** Marie Le Roux, Alain Dupuy, Martine Ropert, Adélie Perrot, Marie Loisel, Louise Gouvrion

**Affiliations:** a Dermatology Department, CHU Rennes, Rennes, France; b Dermatology Department, CHU Rennes, Univ Rennes, Rennes, France; c Department of Biochemistry and Toxicology, CHU Rennes, Elemental Analysis and Metabolism of Metals (AEM2) Platform, Univ Rennes, Rennes, France; d Pathology Department, CHU Rennes, Rennes, France; e Department of Orthopaedic Surgery, CHU Rennes, Rennes University Hospital, Rennes, France

**Keywords:** foreign body granulomatous reaction, metallosis, titanium, vanadium

## Introduction

Metallosis is the release of metallic debris and corrosion products into periarticular tissues secondary to mechanical wear of metal-on-metal components in joint arthroplasty. It is characterized by local inflammatory reaction with fibrosis, tissue necrosis or prosthetic loosening. Although the process is usually confined to the joint and periprosthetic space, rare cases of extracapsular metal particle migration have been described, including cutaneous involvement usually manifesting as skin pigmentation.^1^^,^^2^ Systemic toxicity due to elevated metal ion levels is another possible but uncommon complication.^3^^,^^4^

We report a case of a locoregional and systemic metallosis revealed by the skin, after a reverse shoulder arthroplasty.

## Case report

**Fig 1:**
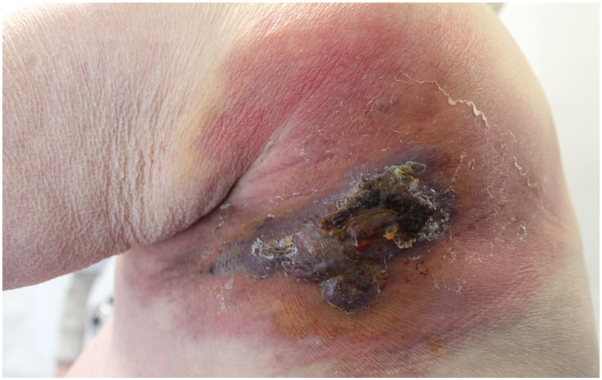
Extensive inflammatory patch over the shoulder centered by a pseudo-tumoral violaceous.

A 76-year-old woman presented in December 2024 with a rapidly growing violaceous tumor surrounded by a large erythematous plaque, on the left shoulder (Fig 1). The patient reported complete functional impairment of the left shoulder for several months.

Her medical history included a left reverse total shoulder arthroplasty in 2022 for glenohumeral osteoarthritis, revised in 2023 for prosthetic instability. The new implant was made of titanium–vanadium– aluminum alloy, hydroxyapatite, polyethylene, and stainless steel.

The differential diagnoses included neoplastic causes (angiosarcoma, lymphoma), as well as inflammatory and infectious diseases.

**Fig 2:**
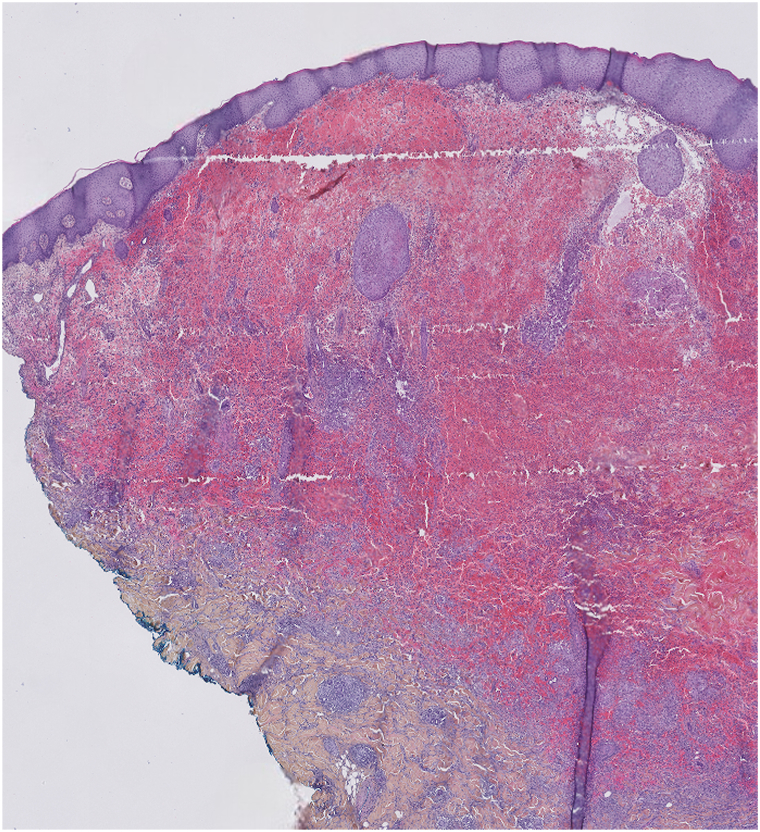
Skin biopsy of the violaceous pseudo-tumoral lesion: showing non-neoplastic interstitial granulomatous reaction (H&E, ×10).

Deep skin biopsies of the lesion revealed a non-neoplastic interstitial granulomatous reaction with ferric deposits highlighted by Perls’ staining (Fig 2). Microbiological cultures (bacterial, mycobacterial, and fungal) were negatives.

**Fig 3:**
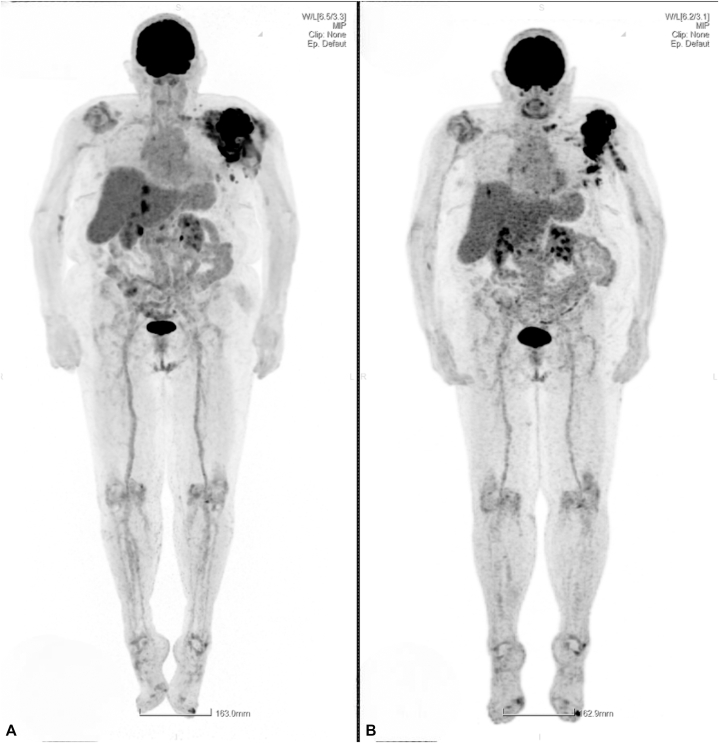
PET-CT scan showing intense hypermetabolism of the left shoulder with multiple necrotic peri-prosthetic collections, hypermetabolic lymphadenopathy and 2 hypermetabolic hepatic lesions.

A positron emission tomography–computed tomography (PET-CT) scan demonstrated intense hypermetabolism of the left shoulder with necrotic peri-prosthetic collections and complete prosthetic loosening. Additional findings included hypermetabolic lymphadenopathy and 2 hypermetabolic hepatic lesions (Fig 3).

Suspecting chronic prosthetic infection, the patient underwent surgical revision. Intraoperatively, greyish collections and complete loosening were noted. All microbiological samples remained sterile. Histopathologic analysis of the joint capsule revealed a marked foreign-body granulomatous reaction with extensive ferric pigment deposits.

Metallosis was then diagnosed.

Quantitative analysis of metals by inductively coupled plasma mass spectrometry (ICP-MS) revealed titanium plasma concentration thirty-fold above normal (34.3 μg/g; ref <1) and skin concentration at 712 μg/g (ref <2 μg/g). Vanadium was also increased, with plasmatic concentration three-fold the normal value (0.46 μg/L; ref <0.14 μg/L) and skin concentration at 28.9 μg/g (hair reference <0.05 μg/g; no skin reference) (Table I). Reference values come from healthy, unexposed populations.^5^

**Table I:** Quantitative monitoring of titanium and vanadium levels by ICP-MS across different sites

	Plasma (μg/L)	M1†	M3‡	Reference	Whole blood (μg/L)	M1	Reference	Skin (μg/g)	Reference	Liver (μg/g)	Reference	Shoulder seroma (μg/g)	Reference
	J0∗				J0			J0		M1		M3	
Titanium	34.3	29.6	30.3	<1	27.9	26.6	<1.4	712	0.5-2	0.22	–	1572	–
Vanadium	0.46	0.41	0.33	<0.14	–	–	–	28.9	<0.05§	<0.1	–	62	–

∗ J0 correspond to the first metal screening after prothesis removal.

† M1 means 1 month after prothesis removal.

‡ M3 means 3 months after prothesis removal.

§ No available reference values in the skin; reference values for hair.

A biopsy of a PET liver hypermetabolic lesion showed non-neoplastic granulomatous inflammation, with a non-elevated titanium concentration (0.22 μg/g; no established hepatic concentration reference).

Skin tests for delayed hypersensitivity to metals were performed on our patient, with readings on day 3 and day 5. They did not reveal any reaction.

Three months after prosthesis removal, the patient developed erythema nodosum with hypercalcemia (2.84 mmol/L), low parathyroid hormone, elevated angiotensin-converting enzyme (80 U/L), and increased 1,25-dihydroxyvitamin D. Skin biopsy of erythema nodosum showed deep foreign body granulomas. Plasmatic titanium remained high (29.6 μg/L). PET-CT revealed persistent glenohumeral hypermetabolism extending to the scapula and active lymphadenopathy (SUVmax 7.6); hepatic lesions had resolved.

These findings were diagnosed as a systemic foreign body granulomatous reaction due to titanium and vanadium.

Five months post-removal, a left shoulder seroma appeared. Aspiration revealed very high titanium (1572 μg/g) with normal vanadium levels. Surgery was deferred, expecting spontaneous resolution.

## Discussion

We report a case of titanium and vanadium metallosis, attributed to chronic prosthetic loosening, which resulted in a generalized and persistant granulomatous reaction.

In cases of metallosis, periprosthetic histology usually shows extracellular black particles, together with intracellular deposits within macrophages, histiocytes, and occasionally multinucleated giant cells, associated with a moderate inflammatory infiltrate.^1^ Granuloma formation is not systematic.^6^

Metal ion levels in blood, skin, and liver were quantified by inductively coupled plasma mass spectrometry (ICP-MS) with a 15-element plasma analysis.

ICP-MS is the reference technique for trace metal quantification, combining a high-temperature argon plasma that ionizes the sample (ICP) with mass spectrometry (MS), which separates and quantifies ions. This method offers high sensitivity, but its availability is limited to specialized toxicology centers. Blood and skin samples can be sent to such centers for analysis.

While Inductively Coupled Plasma Optical Emission Spectrometry (ICP-OES) and Scanning Electron Microscope-Energy Dispersive X-ray spectroscopy (SEM-EDX) are alternative techniques, their sensitivity remains lower.^1^^,^^6^, ^7^, ^8^

Titanium–vanadium alloys are commonly used in shoulder arthroplasty for their biocompatibility and durability. Multiple metal-metal interfaces increase the risk of ion release. Although precise data on titanium clearance remain limited, its mean biological half-life is estimated to be approximately 320 days.^8^ In contrast, vanadium is cleared more rapidly, with reported elimination half-life ranging from 3 to 15 days. This can explain the delayed titanium seroma in our patient 5 months after implant removal.

In most cases, metallosis is usually confined to the joint capsule or around the prosthesis. The corrosion may leads to locoregional extension of the metal debris with skin pigmentation^1^^,^^2^ or granulomatous reaction resulting in pseudotumours.^6^ Prothesis removal is imperative.

In our case, the distant lesions are linked to a systemic granulomatous reaction to foreign body, including lymphadepathy, hepatic nodules and erythema nodosum. Cases of systemic granulomatous foreign-body reaction associated with metallosis remain extremely rare.^9^

Metal ions can also spread throughout the body and cause systemic toxicity.^3^^,^^4^

In our case, plasmatic titanium was thirty times above normal and remained elevated 3 months after prosthesis removal, raising concerns about systemic toxicity. In contrast, the risk of vanadium toxicity was considered lower, as plasmatic concentration were only 3 times above the normal value.

Toxicity of high blood titanium—such as neurologic and renal impairments—have been described.^3^^,^^4^ These findings highlight the need for cautious management and extended monitoring of plasmatic metal levels after prosthesis removal for metallosis.

In our patient, renal function remained normal.

Rare cases suggest that delayed hypersensitivity to titanium could be a distinct pathologic process.^10^ Circulating titanium ions may bind serum proteins, forming hapten-like complexes that trigger immune or hypersensitivity reactions. Patch tests in our patient showed no hypersensitivity.

## Conclusion

Metallosis is a rare complication of arthroplasty. Pigmented or pseudotumorous lesions near prostheses should raise suspicion. In such cases, skin biopsy with metal quantification confirms the diagnosis. Granulomatous reactions may signal systemic spread, and blood metal levels should be monitored after prosthesis removal.

## Conflicts of interest

None disclosed.
